# Geotrichosis Due to Magnusiomyces capitatus: A Severe Emerging Invasive Fungal Disease

**DOI:** 10.7759/cureus.87699

**Published:** 2025-07-10

**Authors:** Fatima Zahra Lfaquir, Abderrahim Chour, Imane Zouaoui, Khalil Zimi, Sarra Aoufi

**Affiliations:** 1 Central Laboratory of Parasitology and Mycology, Ibn Sina University Hospital, Rabat, MAR; 2 Faculty of Medicine and Pharmacy, Ibn Sina University Hospital, Rabat, MAR; 3 Faculty of Medicine and Pharmacy, Mohammed V University, Rabat, MAR

**Keywords:** emerging mycosis, fungal infection, immunocompromised patients, invasive geotrichosis, magnusiomyces capitatus, morocco, north africa, opportunistic fungi

## Abstract

Geotrichosis is a rare opportunistic mycosis caused by emerging yeasts rarely associated with fungemia. We report a case of invasive *Magnusiomyces capitatus* (*M. capitatus*)* *infection in a neutropenic patient. A 71-year-old man with rectosigmoid cancer undergoing chemotherapy was admitted to the emergency department with septic shock. Laboratory tests revealed a severe inflammatory response, agranulocytosis, and acute renal failure requiring hemodialysis via a femoral catheter. An infectious workup performed at the Central Parasitology and Mycology Laboratory of Ibn Sina University Hospital in Rabat included blood cultures, urine samples, hemodialysis catheter samples, and a protected distal specimen (PDS). Empirical broad-spectrum antibiotic therapy was initiated, followed by caspofungin due to a lack of clinical improvement. Microscopic examination of urine, catheter, and PDS specimens revealed arthrospores. Blood cultures turned positive after 20 hours of incubation. Cultures on Sabouraud agar showed whitish, dry colonies with irregular edges, typical of *Geotrichum* spp. Precise identification of *M. capitatus* was achieved using matrix-assisted laser desorption ionization-time-of-flight mass spectrometry (MALDI-TOF). Despite appropriate antifungal treatment, the patient died 10 days after admission. This case underlines the severity of invasive *M. capitatus* infections, often overlooked in immunocompromised patients, and highlights the critical importance of rapid and accurate diagnosis with modern tools like mass spectrometry to optimize management.

## Introduction

Geotrichosis is a rare opportunistic fungal infection caused by emerging yeasts belonging to the genus *Geotrichum*. This genus was recently reclassified as *Magnusiomyces capitatus *(*M. capitatus*)* *(formerly *Geotrichum capitatum*). These yeasts are ubiquitous in the environment and form part of the normal microbiota of the respiratory and gastrointestinal tracts. It is believed that infection primarily occurs through inhalation or translocation from the gut in immunocompromised patients [1]. Although *M. capitatus*-caused invasive infections are increasingly recognized as emerging pathogens, they remain uncommon and poorly understood in clinical practice [2]. These infections primarily affect immunocompromised patients, especially those undergoing intensive treatments such as chemotherapy. This population is at a higher risk for severe complications.

Furthermore,* M. capitatus *exhibits intrinsic resistance to certain antifungal agents, particularly echinocandins, which are commonly used to treat invasive fungal infections. This resistance complicates therapeutic management significantly and may lead to increased morbidity and mortality associated with these infections [3]. Therefore, improving our understanding of the clinical, microbiological, and therapeutic characteristics of this emerging mycosis is essential to enabling earlier diagnoses and tailoring antifungal strategies to optimize patient outcomes. This study reports a case of invasive geotrichosis caused by *M. capitatus *in a neutropenic patient with rectosigmoid carcinoma undergoing chemotherapy.

## Case presentation

This 71-year-old patient has no prior medical history and is being treated for rectosigmoid carcinoma. He was receiving chemotherapy according to the FOLFIRINOX protocol, which included oxaliplatin (85 mg/m²), irinotecan (180 mg/m²), leucovorin (400 mg/m²), followed by a 5-fluorouracil bolus (400 mg/m²) and a continuous infusion (2400 mg/m² over 46 hours), administered every two weeks. One month after beginning treatment, the patient was admitted to the emergency department in septic shock. He presented with a fever, persistent hypotension despite fluid resuscitation, tachycardia, and altered mental status. Laboratory tests revealed severe inflammation, agranulocytosis, thrombocytopenia, and acute renal failure requiring hemodialysis (Table 1).

**Table 1:** Patient’s biochemical and hematological results

Parameter	Patient result	Normal range/reference values
C-reactive protein (CRP)	492 mg/L	<5 mg/L
Creatinine	28 mg/L	7.2-12.5 mg/L
Urea	0.12 g/L	0.03-0.07 g/L
Sodium (Na⁺)	140 mEq/L	135-145 mEq/L
Potassium (K⁺)	5.8 mEq/L	3.5-5.1 mEq/L
Chlorides (Cl⁻)	105 mEq/L	98-107 mEq/L
Bicarbonates (HCO₃⁻)	18 mEq/L	22-28 mEq/L
Neutrophils (agranulocytosis)	0.4 × 10³/μL	6-26 × 10³/μL
Platelets (thrombocytopenia)	6 G/L	150-450 G/L
Hemoglobin	8 g/dL	13.4-19.8 g/dL

**Figure 1:**
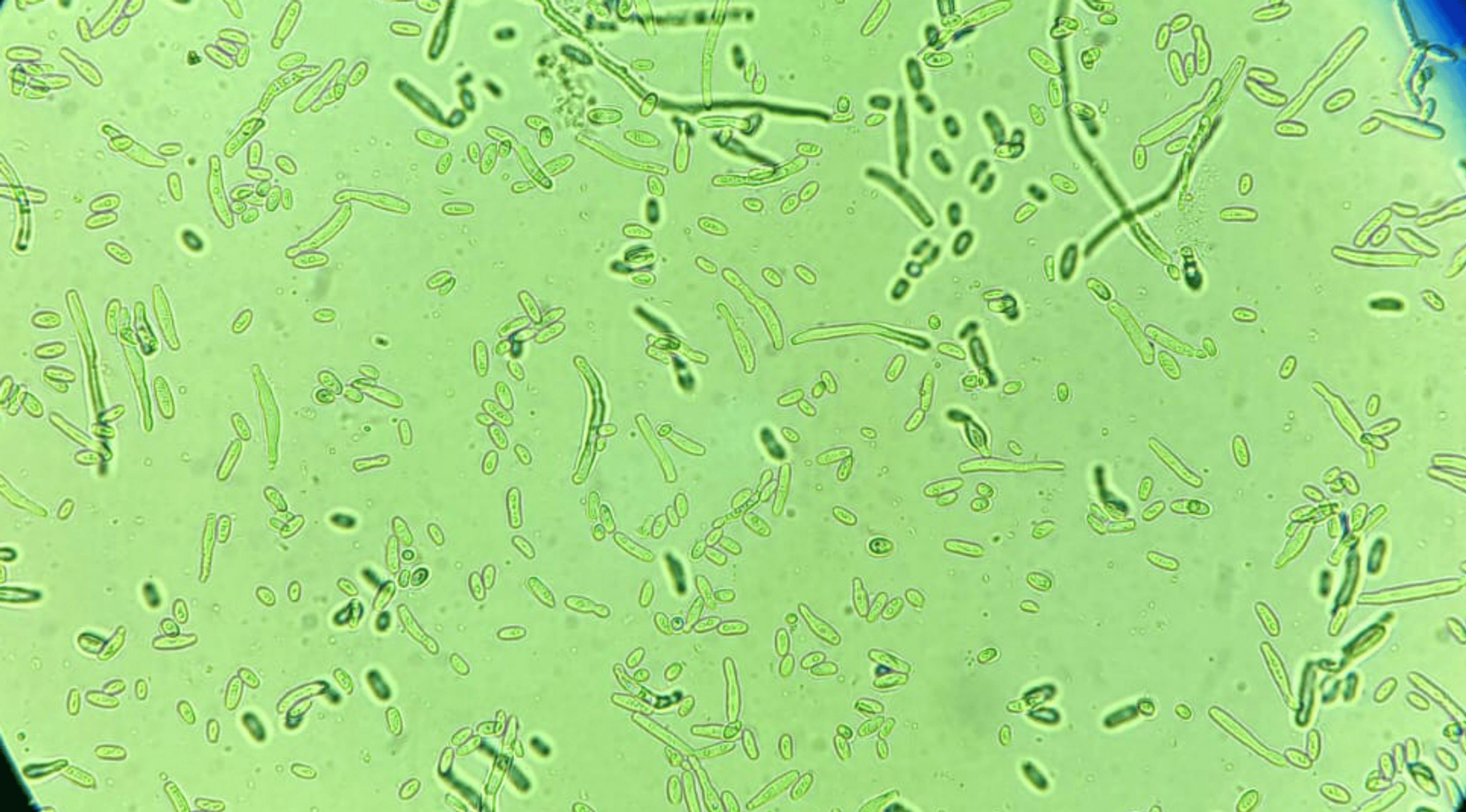
Microscopic view of the arthrospores of Geotrichum spp. after culture at 40x magnification

Based on the clinical and laboratory findings, including blood cultures, urine specimens, hemodialysis catheter samples, and a protected distal specimen (PDS), an infectious disease assessment was carried out. The samples were sent to the Central Parasitology and Mycology Laboratory at Ibn Sina University Hospital in Rabat. Broad-spectrum antibiotic therapy was promptly initiated. However, due to the absence of clinical improvement, fungemia was suspected, and antifungal treatment with caspofungin was initiated, administered intravenously at a dose of 70 mg per day. Microscopic examination of the urine, the hemodialysis catheter, and the PDS revealed filaments fragmenting into arthrospores at 40x magnification. Blood cultures were positive after 20 hours of incubation. After 24 hours of incubation in aerobic conditions at 37°C on Sabouraud-Chloramphenicol and Sabouraud-Cycloheximide media, cultures of all samples showed whitish colonies with a dry appearance and irregular contours. Microscopic examination of the cultures revealed filaments with arthrospores, indicating* Geotrichum spp*. (Figure 1).

Mass spectrometry (matrix-assisted laser desorption/ionization time-of-flight (MALDI-TOF)) revealed that the fungal agent was precisely identified as *M. capitatus*. Despite intensive voriconazole treatment, the patient's condition deteriorated. The patient died 10 days after being admitted to the hospital.

## Discussion

*M. capitatus *is a cosmopolitan, opportunistic yeast that is saprophytic in the external environment and commensal in the digestive, respiratory, and skin tracts of humans. It can cause serious invasive infections, primarily in immunocompromised patients [4,5].

Systemic infections with *M. capitatus* in immunocompromised patients are less common than invasive candidiasis and aspergillosis [6]. Similar risk factors have been identified, including profound neutropenia secondary to chemotherapy, dysbiosis due to prophylactic antibiotic use, and the presence of invasive devices such as catheters [4,5,7]. Our patient had all these risk factors: he had a solid tumor and was receiving hematotoxic chemotherapy, which induced profound neutropenia. He was also receiving broad-spectrum antibiotic therapy and had a hemodialysis catheter.

The clinical manifestations of invasive geotrichosis generally resemble those of other invasive fungal infections, particularly candidiasis. The infection most often begins with an acute fever that does not respond to antibiotics. It usually progresses rapidly, leading to multiple organ failure despite appropriate antifungal treatment [8].

In our patient's case, the persistence of fever despite the initiation of broad-spectrum antibiotic therapy strongly suggested an invasive fungal infection, justifying treatment with caspofungin at a dose of 70 mg intravenously.

The diagnosis of invasive geotrichosis is primarily based on blood culture, which remains the gold standard for detecting the spread of this fungus. According to data from the literature, 75% of disseminated *M. capitatus *infections are diagnosed by blood culture [9]. In our case, blood cultures also proved positive within 24 hours. 

*M. capitatus* was detected in several locations, including the urine, the hemodialysis catheter, and the PDS. This is consistent with data from the literature, where approximately 43% of cases involved more than one site [9]. The fact that the fungus was isolated from multiple sites suggests hematogenous spread of the infection.

The treatment of invasive* M. capitatus* infections is complicated by their rarity and the fact that they are often confused with invasive candidiasis due to their similar clinical presentation. This similarity can lead to diagnostic errors and inappropriate initial treatment choices. Indeed, several studies have shown that all *M. capitatus* isolates have high minimum inhibitory concentrations (MICs) for echinocandins, revealing intrinsic resistance to this class of antifungal agents [3]. Combined with the lack of official treatment recommendations and standardised sensitivity thresholds, this resistance makes management difficult and treatment responses highly variable, highlighting the need for an individualised therapeutic approach [10]. Furthermore, current recommendations advocate empirical antifungal treatment with caspofungin or lipid amphotericin B for patients with fever and neutropenia. In our case, treatment with caspofungin was initially initiated due to suspicion of invasive candidiasis, but it proved ineffective.

Invasive *M. capitatus* infections are associated with a poor prognosis due to nonspecific clinical signs and diagnostic difficulties. The outcome is particularly serious in patients with severe neutropenia. Despite the use of antifungal treatments such as liposomal amphotericin B, itraconazole, or flucytosine, mortality can reach 90% [11]. In our case, the patient died 10 days after admission despite intensive treatment with voriconazole.

## Conclusions

The presented case underscores the severity of *M. capitatus *infections in immunocompromised patients and highlights the importance of rapid, accurate diagnosis and therapeutic management. Clinicians must be vigilant about the emergence of these mycoses, especially in immunocompromised individuals. Furthermore, rapid and reliable identification of fungi, notably through techniques such as mass spectrometry, is essential to selecting appropriate antifungal therapy. This will reduce associated mortality and optimize the management of invasive fungal infections.
